# Supplementary material for: Self-Assembling Tacrolimus Nanomicelles for Retinal Drug Delivery
Source: Pharmaceutics. 2020 Nov 10;12(11):1072. doi: 10.3390/pharmaceutics12111072 (PMC7698121; doi:10.3390/pharmaceutics12111072)
Supplement: Supplementary file 1 [file pharmaceutics-12-01072-s001.pdf]

Supplementary Materials: Self-Assembling Tacrolimus Nanomicelles for Retinal Drug Delivery

Vrinda Gote, Abhirup Mandal, Meshal Alshamrani and Dhananjay Pal

1. Methods

LDH Assay

Lactose Dehydrogenase (LDH) is a constituent of many cells. When the cell membrane is damaged, LDH is released into the surrounding media. The amount of LDH release can be correlated with the percentage of cell death. D407, CCL 20.2 and RF/6A cells were seeded in 96-well plate at a density of 1 × 10^4 cells/well. Culture plates were incubated in an incubator containing 5% CO_2 at 37 °C for 24 h. Samples of NMF, TAC-NMF and TAC solution were prepared in SFM the same concentration range as previously described. The cells were treated with the samples for 24 h under incubation. At the end of the treatment period, the plates were carefully centrifuged at 20,000 r.p.m. for 10 min. The supernatant SFM was separated from the cells and added to a new 96-well plate. LDH assay kit (Takara Bio Inc., Shiga, Japan) was utilized for the detection of LDH released into the media. Absorbance of red formazan crystals was measured spectrophotometrically using a micro plate reader at an excitation wavelength of 490 nm. Similar to the MTT assay, the positive control was Triton-X 5% and the negative control was SFM. The amount of LDH release was determined using the following formula:

$$\text{LDH release}\,(\%) = \frac{\text{Absorbance of sample - absorbance of negative control}}{\text{Absorbance of positive control - absorbance of negative control}} \times 100 \quad (6)$$

2. Results

LDH Assay

To confirm the results obtained by MTT assay, LDH assay was also performed. Drug-free NMF, TAC and TAC-NMF formulations were analyzed for cytotoxic effect on D407, CCL 20.2 and RF/6A cells after 24 h of treatment. In response to cytotoxic agents, cellular death by necrosis or apoptosis release of the intracellular LDH catalytic enzyme into the extracellular medium may occur. LDH released in the extracellular space is proportional to cell death and can be quantified by colorimetric LDH assay [51]. Figure 4 A-D the percentage of LDH released in the medium after 24-h treatment with drug-free NMF (Figure 4A) in D407, CCL 20.2 and RF/6A cells. Percentage of LDH released after 24 h of treatment with TAC and TAC-NMF in D407, CCL 20.2 and RF/6A can be seen in Figures 4B, 4C and 4D. Polymers in the nanomicelles interact with the lipid bilayer of the cells and enter the cells through endocytosis [52, 53]. This can potentially damage the cell membrane and intracellular enzyme lactase dehydrogenase can be released into the external media. It can be seen that in Figure 2A, the percentage of LDH released into the media is very low and comparable to the negative control, serum free medium. This implies that the mixed polymeric nanomicelles of HCO-40 and OC-40 amphiphilic surfactant-based polymers demonstrate negligible cytotoxicity to ocular cell lines like D407, CCL 20.2 and RF/6A (Figures 6A and 7A). In Figures 7B–D, the LDH release was significantly higher for TAC solution alone as compared to TAC-NMF solution. Moreover, the percentage of LDH release increased with increasing concentration of TAC and TAC-NMF treatment groups. This demonstrates the dose dependent cytotoxicity of TAC and TAC-NMF on D407, CCL 20.2 and RF/6A cells.

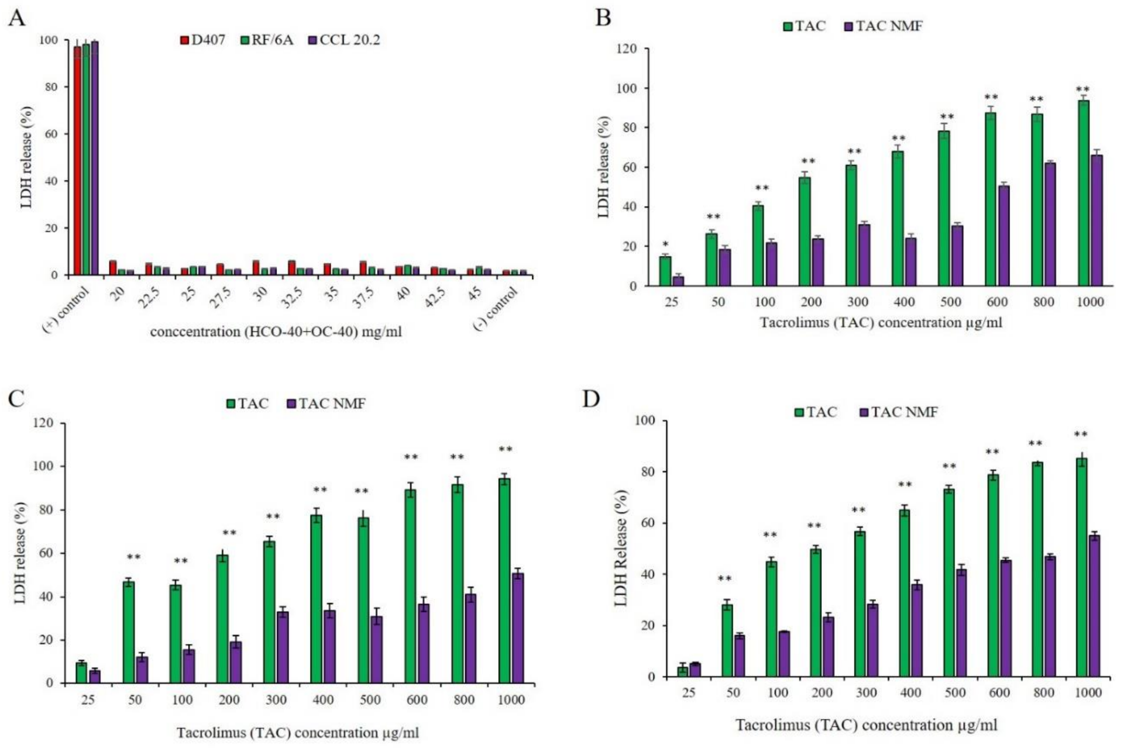

Figure S1. In-vitro cytotoxicity determination by LDH Assay. (**A**) LDH (%) of D407, CCL 20.2 and RF/6A cells after treatment with placebo NMF composed of mixture of HCO-40 and OC-40 amphipylic polymers ranging from 20–42.5 mg/mL of total HCO-40 and OC-40 in a fixed ratio of (HCO-40:OC-40: 3.5:1) for 24 h. (**B**) Cell Viability (%) of retinal pigment epithelium cells (D407) after treatment with TAC and TAC-NMF for 24 h. (**C**) Cell viability (%) of conjunctival cells (CCL 20.2) after treatment with TAC and TAC-NMF for 24 h. (**D**) Cell viability (%) of choroidal endothelial cells (RF/6A) after treatment with TAC and TAC-NMF for 24 h. (* $p \leq 0.05$ and ** $p \leq 0.01$ as compared to the corresponding control group, here TAC).

© 2020 by the authors. Licensee MDPI, Basel, Switzerland. This article is an open access article distributed under the terms and conditions of the Creative Commons Attribution (CC BY) license (http://creativecommons.org/licenses/by/4.0/).
